# Promoting physical activity among community groups of older women in socio-economically disadvantaged areas: randomised feasibility study

**DOI:** 10.1186/s13063-019-3312-9

**Published:** 2019-04-25

**Authors:** Emma R. Lawlor, Margaret E. Cupples, Michael Donnelly, Mark A. Tully

**Affiliations:** 1 UKCRC Centre of Excellence for Public Health (Northern Ireland), School of Medicine, Dentistry and Biomedical Sciences, Queen’s University Belfast, Clinical Sciences Block B, Royal Victoria Hospital, Belfast, BT12 6BA Northern Ireland, UK; 2 Department of General Practice and Primary Care, Queen’s University Belfast, 1 Dunluce Avenue, Belfast, BT9 7HR Northern Ireland, UK; 3 School of Health Sciences, Institute of Mental Health Sciences, Ulster University, Shore Road, Newtownabbey, BT37 0QB Northern Ireland, UK

**Keywords:** Community-based, Older women, Socio-economically disadvantaged, Physical activity, Intervention, Social networks

## Abstract

**Background:**

Insufficient physical activity (PA) is a major public health issue. Whilst PA is an important contributor to disease prevention, engagement in PA decreases with age, particularly among women in socio-economically disadvantaged areas. Research using existing support networks to engage ‘hard to reach’ populations in PA interventions is sparse. We developed and tested the feasibility of a PA-promoting intervention for older women within existing community groups in socio-economically disadvantaged areas.

**Methods:**

The Medical Research Council guidelines for complex interventions were used to guide the intervention’s development. We recruited participants (*n* = 40) from older (aged ≥50 years) women’s groups from four different community centres. A 12-week programme was delivered during existing sessions, informed by Social Practice Theory. The sessions provided education about PA, social support in the form of a PA ‘buddy’, group discussion and follow-up telephone calls, as well as printed information about local opportunities to participate in PA. The main uncertainties tested were rates of participant recruitment, retention, and completion of assessments of PA by accelerometry and of mental health using the Hospital Anxiety and Depression Scale (HADS). Intervention acceptability was assessed by questionnaire, and focus group interviews elicited participants’ views about the intervention. Qualitative data were subjected to framework analysis.

**Results:**

The recruitment rate was high; 87% (*n* = 40/46) of women consented to participate, and 78% (*n* = 31) attended all education sessions. Uptake of follow-up telephone calls and PA ‘buddies’ was low. Few participants provided valid accelerometer data, but 63% (n=25) completed the HADS questionnaire at all time points. The printed materials and education sessions were viewed positively; telephone calls and ‘buddy’ support were not valued. Participants believed that organised group activities would lead to increased PA engagement, and whilst participants disliked wearing a waist accelerometer, they thought that regular PA feedback would facilitate necessary goal-setting.

**Conclusions:**

High recruitment and retention rates suggest that use of existing social support groups is an acceptable and attractive method of delivering a PA intervention to this population. A randomised controlled trial of the intervention appears feasible, but its design requires refinement of the social support component, facilitation of goal-setting and reconsideration of the assessment of PA.

**Trial registration:**

ClinicalTrials.gov, NCT02880449. Registered on 26 August 2016.

**Electronic supplementary material:**

The online version of this article (10.1186/s13063-019-3312-9) contains supplementary material, which is available to authorized users.

## Background

Physical inactivity is the fourth leading risk factor for global mortality, contributing to 9% of deaths worldwide annually [1], and is associated with large social and economic consequences, including high direct health-care costs, productivity losses, and disability-adjusted life-years [2, 3]. However, despite strong evidence for the role of physical activity (PA) in the prevention of cardiovascular disease (CVD), cancer, type 2 diabetes mellitus and premature mortality [1, 4, 5], 39% of adults (20 million people) [6] within the United Kingdom are still not achieving the current Chief Medical Officers’ recommendations for 150 min of PA per week [7].

Research suggests that some population sub-groups are particularly at risk of being inactive. The number of older people is increasing, with this group often facing unique and multiple challenges to health [8–10]. Despite the health and psychosocial benefits of PA for older people being well recognised [4, 5, 10–13], their engagement in PA is often low [14]. In addition, individuals living in socio-economically disadvantaged areas typically have lower levels of participation in PA than those in higher socio-economic groups [11, 15], further increasing health inequalities [16]. Furthermore, females are less likely to engage in PA than men, with 68.6% of females being physically inactive in comparison to 58.0% of males in the United Kingdom [6, 14]. Consequently, older women living in socio-economically disadvantaged groups are a population sub-group needing immediate attention to increase PA, and innovative approaches are imperative to engage this group in PA promotion services.

Although PA promotion programmes are available, many individuals fail to engage in them due to lack of time, access issues, competing commitments, inconvenient programme scheduling, financial costs and lack of affiliation with others in the programme [17–21]. Programmes that are context-specific and delivered within the community may help overcome some of these barriers [22], such as accessibility, convenience and cost. This may in turn potentially increase participant recruitment and retention, which is particularly problematic within socio-economically disadvantaged communities [23]. Previous community-based PA promotion interventions [24, 25] delivered in a variety of non-medical settings, have been found to be acceptable and produce beneficial health outcomes. However, these studies often recruit participants who have not had any previous contact with each other; little is known about the effect of recruiting participants and delivering community-based interventions within pre-existing social groups.

Previously, peer support interventions targeting PA have produced beneficial effects on a number of health-related outcomes [26–28] and increased engagement in PA [11, 27, 29]. Building on this evidence, an innovative approach, delivering a peer support intervention within a pre-existing group of people may avoid the apprehension associated with joining new groups, enhance their enjoyment of the programme and increase the likelihood of sustained behaviour change as the group could provide ongoing social support [30–32]. Tapping into pre-existing groups and venues already used by the target population is an under-used strategy that is a more feasible and sustainable approach than establishing new groups, and warrants further research [33].

Community centres tend to be based in socio-economically disadvantaged areas, hence being perfectly placed to deliver a context-sensitive health promotion intervention in close vicinity to those experiencing the worst health [34, 35]. They provide a unique opportunity to gain access to ‘hard to reach’ sub-populations because many of these groups already use the centres. In particular, they traditionally regularly host older women’s groups; these pre-existing social groups present an opportunity to deliver a low-cost PA intervention to inactive older women.

By following the Medical Research Council (MRC) guidelines for the development and evaluation of complex interventions [36], we developed a community-based intervention promoting PA for older women (aged ≥50 years) in socio-economically disadvantaged areas, using evidence from a systematic review and input from service providers and users, and tested the feasibility of the intervention’s delivery and evaluation in a randomised trial. The programme was delivered within the usual sessions, providing education about PA, encouragement for group and one-to-one social support, and information about local opportunities for PA and walking routes.

The objectives of this feasibility study were to (1) test the operational aspects of the trial design in terms of recruitment, retention and outcome assessments; (2) ensure that the proposed methodological approach was feasible for a large-scale trial [36–39]; and (3) gather participants’ views of the acceptability of the intervention and trial design [37]. These were determined by assessing the rates of participant recruitment, retention and completion of PA and mental health outcome measures, and by exploring participants’ views of the intervention and the research methods through qualitative interviews.

### Ethical approval

The Queen’s University Belfast Research Ethical Committee approved the study on 3rd August 2016 (application no. 16.38v2), and the trial was registered with ClinicalTrials.gov (identifier NCT02880449).

## Methods

### Participants and study setting

We identified four community centres located in socio-economically disadvantaged areas of Belfast, Northern Ireland. From each centre, one older women’s group (aged ≥50 years) that would usually engage in activities such as crafts, day trips and other social opportunities was invited to participate; all four agreed. According to the Northern Ireland Multiple Deprivation Measure 2010 [40], the centres were located in the 25% most socio-economically disadvantaged areas in Northern Ireland, with two in the 10% most socio-economically disadvantaged areas, and all were in urban residential areas. Three centres were situated near to at least one park; one was in an area with many hills and had no proximity to parks. All centres were within a 15-min walking distance of a leisure centre.

All members of the four groups (*n* = 46) were invited to participate in the study by ERL at one of their regular meetings. They were provided with an information sheet and an opportunity to ask questions. No exclusion criteria were applied: the women were informed that participation was voluntary and they could withdraw at any time. Written consent was gained. As this was a feasibility study, there was no formal sample size calculation, but it was anticipated that 40 participants would provide sufficient information to meet our objectives.

### Data collection

The key uncertainties to be examined were the rates of recruitment, retention and completion of outcome measures. The numbers that attended each education session, completed review assessments and accepted telephone calls were recorded. Reasons for drop-out were recorded if provided by the participant.

In addition, participants were asked to wear a validated ActiGraph GT3X waist-worn accelerometer (ActiGraph, Pensacola, FL, USA) over 7 days (to provide an objective measure of participation in PA) and to complete the Hospital Anxiety and Depression Scale (HADS) [41] (a validated mental health measure). These outcome assessments were chosen as the intervention is focused on increasing PA and providing social support, which may be expected have an impact on mental health. The completion rates of these were recorded at baseline, 6 weeks, 12 weeks and 6 months after baseline in order to test the feasibility of their use in a definitive trial. Criteria to determine feasibility were recruitment of at least 80% of each group, with 75% retention of recruited participants and 60% completion of proposed assessment measures.

To determine the acceptability of the programme, participants were asked to rate their experience of the study using a post-study exit questionnaire at 6 months from baseline. Further, all participants were invited to participate in an end-of-study focus group using a semi-structured interview schedule (*see* Additional file 1) in which their views of the feasibility and acceptability of the intervention, components felt to be least or most beneficial, and improvements for a future study were explored. Focus groups took place in each group’s own community centre either before or after a scheduled group session, dependent on each group’s preference.

### Intervention development

This intervention and its mode of delivery was designed by (1) identifying existing evidence, (2) identifying and developing theory, and (3) modelling the process and outcomes [36]. Evidence used in the intervention development was identified by (1) a systematic review investigating the effectiveness of community-based interventions for the secondary prevention of CVD on behavioural risk factors [42] and (2) qualitative analysis of interviews with stakeholders involved in the development or delivery of community-based health promotion interventions. This enabled an understanding of the problem, and identification of an appropriate population group and behaviour to be targeted by an intervention.

Salient findings from our systematic review [42] and stakeholder interviews included the identification of a dearth of community-based health promotion interventions targeting women and socio-economically disadvantaged communities, and lack of detail on intervention content and theoretical framework. We found that effective interventions are often individually tailored, use social support and regular facilitator contact, and can increase PA, although more information about objective PA measurement is required. We also identified the potential of using pre-existing groups for successful recruitment to and delivery of health promotion interventions.

The theoretical framework utilised in an intervention can help to identify the processes and sources of influence in behaviour change, and it can increase the likelihood of developing an effective intervention [36, 43]. Our initial review work identified evidence of the importance of social elements in behaviour change and led us to base our intervention on Social Practice Theory (SPT) [44–46], which enables behaviours to be considered as a social issue rather than focusing on individuals’ attitudes, behaviours and choices [47, 48]. SPT highlights the need for relevant information and acquisition of appropriate skills to implement behaviour change, identifying three interactive elements (materials, meanings and competencies) which facilitate behaviour change. ‘Materials’ refers to ‘things, technologies, tangible physical entities, and the stuff of which objects are made’ ([45] p. 14). ‘Meanings’ refers to understandings about significance, shared amongst a group, and is specifically directed towards a behaviour or thing [49]. ‘Competencies’ encompasses ‘skill, know-how and technique’ ([45] p. 14). All three elements must exist for the performance of the behaviour. Table 1 shows how our intervention components relate to these elements.

**Table 1:** Description of intervention components

Elements of Social Practice Theory (SPT)	Behaviour change technique (BCT) labels	BCT groups	Intervention components
Materials	3.2 Social support (practical)	Social support	Encourage identification of a ‘buddy’ (e.g., group member, friend, relative) to provide support for physical activity (PA) Group feedback and discussion on methods to increase PA
	3.3 Social support (emotional)	Social support	‘Buddy’ to provide peer support to increase PA Group to provide encouragement to other members to increase PA Researcher to provide encouragement for PA during group sessions and telephone calls
	4.1 Instruction on how to perform a behaviour	Shaping knowledge	Education sessions to include advice on different methods to increase PA Distribute to participants the printed brochures and maps
Meanings	5.1 Information about health consequences	Natural consequences	Education sessions to include information on health benefits of PA and harms of lack of PA
	5.3 Information about social and environmental consequences	Natural consequences	Education sessions include information on environmental benefits of active travel
	5.6 Information about emotional consequences	Natural consequences	Education sessions to include information on PA benefits for mental health and stress
	13.1 Identification of self as role model	Identity	Inform participants during education sessions that they could be role model to family members and others in group
Competencies	1.2 Problem solving	Goals and planning	Telephone calls with researcher to discuss and provide solutions to barriers to engagement in PA encountered by participants Group feedback and discussion to discuss barriers to engagement in PA encountered and suggest solutions
	4.1 Instruction on how to perform a behaviour	Shaping knowledge	Education sessions to include advice on different behaviours to increase PA

Intervention components are mapped onto relevant behaviour change techniques (Michie et al., 2013 [43]) and the elements of Social Practice Theory (Shove et al. 2012 [45])

Lack of detail regarding behaviour change interventions can lead to challenges in reliably reproducing interventions and synthesising findings within systematic reviews [36, 43, 50–52]. The use of Michie et al.’s [43] Behaviour Change Taxonomy helps to address these factors and to investigate causal pathways and mechanisms of change [43, 53]. The content of our intervention programme was mapped onto specific behaviour change techniques (BCTs): social support, shaping knowledge, natural consequences, identity, and goals and planning (Table 1). These BCTs were selected on the basis of findings of our systematic review [42] regarding effective intervention components and of stakeholder interviews.

**Fig. 1:**
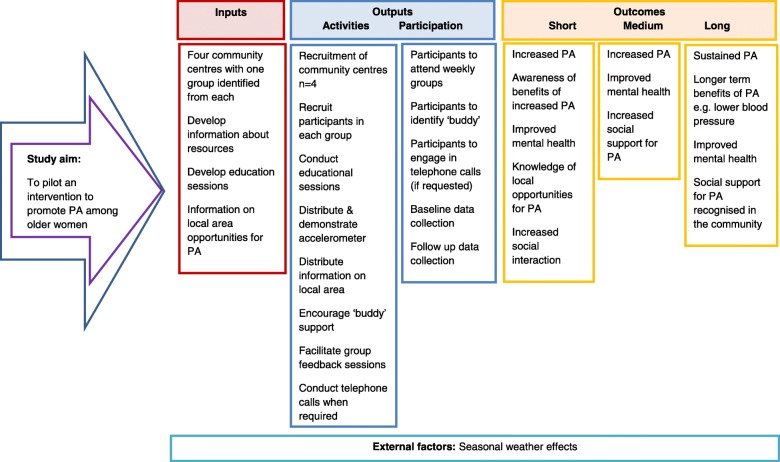
Logic model of intervention

Finally, input from an experienced staff member at one of the community centres, who was not involved in intervention delivery or data collection, was sought to ensure that the intervention would be acceptable and context-sensitive, and would address participants’ needs. This input identified a need for weekly education sessions, informal delivery and integration with the existing group format. These findings were used in constructing a logic model (Fig. 1).

### Intervention

The resulting intervention consisted of three face-to-face group education sessions, encouragement to enlist the support of a buddy (e.g., spouse, partner, friend, or group member), an information pack and the option of weekly telephone contact. Each education session lasted approximately 20 min. It was planned that sessions would be held during three successive weekly meetings of the pre-existing groups, but, due to groups having other commitments, occasionally sessions were postponed by a week. At each session, participants had the opportunity to share their experiences with the group, capitalising on the opportunity to gain social support (Table 2).

**Table 2:** Overview of intervention content to be delivered in each of three education sessions

Education Session	Core components with instructions regarding delivery by researcher
Session 1: Introduction to PA	Examples of physical activity (PA): Ask group for examples and types of PA they do, to stimulate conversation and engage. Elaborate on ideas suggested by participants. Provide information on benefits of walking, green gyms, gardening, dancing, cycling and housework.
	PA recommendations: Ask group if they are aware of any recommended PA guidelines. Discuss guidelines; give suggestions to help achieve them (e.g., brisk walking, carry shopping). Emphasise importance of improvements being individual to the person.
	Health and other benefits of PA: Ask group if they know of any health or other benefits of PA not already discussed. Elaborate on ideas suggested by participants. Ensure discussion of examples, including improving balance, reducing stress, lessening chronic disease, building friendships, helping environment, being a role model.
	Safety: Discuss importance of doing activities the participant feels confident in, right equipment and safety.
	Opportunity to ask researcher questions; opportunity for group discussion.
Session 2: How to increase PA and make PA enjoyable	Progress review: Ask group if they have engaged in any PA during past week, their barriers to doing PA and how they overcame these. Allow opportunity for group discussion.
	Specify ways to get active: Suggest easy ways to increase PA (e.g., get off at earlier bus stop, use stairs instead of lift).
	How to make PA more enjoyable: Provide suggestions (e.g., set targets, go on family day trips, walk with a friend).
	Information on local area: Discuss information provided in information packs. Remind of importance of safety.
	Opportunity to ask researcher questions; opportunity for group discussion.
Session 3: The importance of social support and how to maintain PA in the long term	Progress review: Ask group if they have engaged in any PA during the past week, any barriers to engaging in PA, how they overcame these. Allow opportunity for group discussion.
	Social support: Discuss how social support helps PA. Suggest work towards goals together, try new activities.
	Encourage participants to identify a group member, friend or spouse for mutual support for PA.
	Maintenance of PA: Stress importance of PA for the long term. Provide suggestions (e.g., make it part of their routine, do things they enjoy, do short sessions regularly). Remind of importance of safety.
	Follow-up: Researcher states that they will be back in 3 weeks to conduct follow-up assessments. Offer all a weekly phone call to provide encouragement and problem solving during the 3 weeks.
	Opportunity to ask researcher questions; opportunity for group discussion.

The information pack included information generated by the researcher ERL and adapted from already existing resources, with input from participants during the recruitment session. It consisted of printed materials, including maps of naturally occurring routes (e.g., road loops or footpaths) and parks as well as information on local PA opportunities, including groups currently ongoing in local facilities. The intervention was delivered between September 2016 and June 2017.

### Study design

**Fig. 2:**
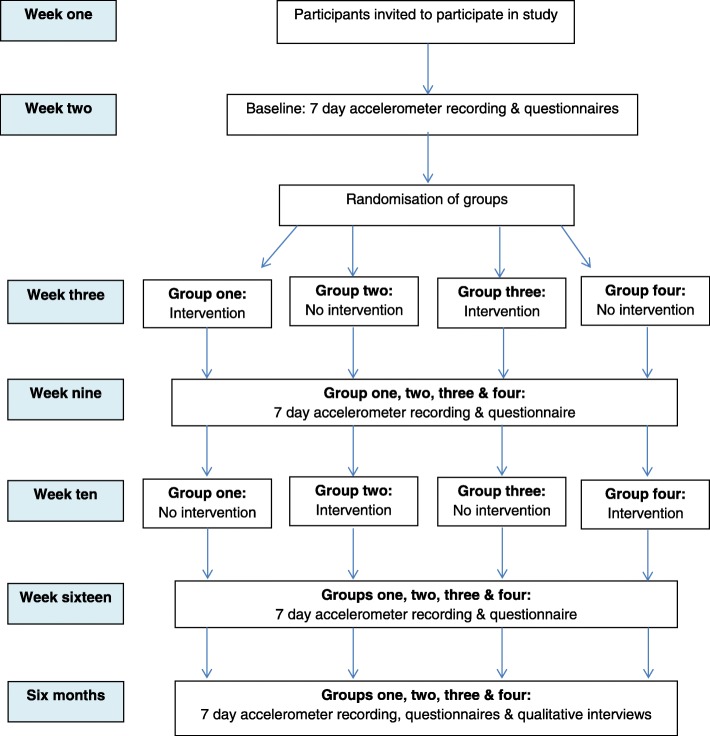
Flow diagram of intervention timeline

A parallel-group delayed intervention design, with two study arms, was used to allow involvement of all participants. At baseline, all centres received information about the study. Two centres received the intervention from week 1 for 6 weeks (immediate intervention group), and two centres received the intervention at week 7 for 6 weeks (delayed intervention group) (Fig. 2). At baseline, participants in the delayed intervention were given information about the study but received no further research contact in the following 6 weeks. No change to the methodology was made after trial commencement.

### Randomisation and blinding

Randomisation to allocate community centres to either the immediate or delayed intervention group was conducted using a computerised random sequence generator (www.random.org). To avoid contamination, randomisation was conducted at the community group level by MEC, who was not involved in the intervention delivery. Allocations were placed in sealed, opaque envelopes until baseline measurements were completed. Due to the nature of the intervention, it was not possible to blind the researcher (ERL) or participants to their allocation.

### Qualitative methods

All participants of the study were invited to participate in a focus group held in their own local community centre at their 6-month follow-up session, with the aim of exploring participants’ views of the outcome assessments, programme components and suggestions for refinement of the intervention and research methods for a future definitive trial. Using a semi-structured interview schedule (Additional file 1), focus groups were conducted, recorded and transcribed verbatim by ERL. Qualitative findings were analysed using a framework analysis, with ERL and MAT independently identifying codes to ensure rigor, and meeting to discuss the appropriateness of the codes and to generate themes using an iterative process. Anonymised quotes provide supportive evidence of the analysis. Data were managed and stored using NVivo software (version 11, 2015; QSR International Pty Ltd., Doncaster, Australia).

## Results

### Participant demographics

The sample consisted of 40 participants, with group sizes ranging from 9 to 12 participants (Table 3). Almost two-thirds of participants were aged 65 years or older (*n* = 25; 62.5%), and over half were separated, widowed or divorced (*n* = 23; 57.5%). Almost one-third had no access to a vehicle for transport (*n* = 13; 32.5%), and over two-thirds (*n* = 27; 67.5%) reported having a long-term illness limiting engagement in PA. The most commonly reported examples of PA participants engaged in were walking (*n* = 28; 70.0%), swimming (*n* = 8; 20.0%) and water aerobics (*n* = 4; 10.0%). Few participants reported engaging in PA at least once per month with a family member (*n* = 11; 27.5%) or friend (*n* = 16; 40.0%). There were minor differences in baseline characteristics between groups related to age, marital status and vehicle access.

**Table 3:** Baseline participant demographic information (N = 40)

Variable		Immediate intervention			Delayed intervention			
		Group 1 (n = 9)	Group 2 (n = 10)	Total (*n* = 19)	Group 3 (n = 12)	Group 4 (n = 9)	Total (n = 21)	Total (n = 40)
		n (%)	n (%)	n (%)	n (%)	n (%)	n (%)	n (%)
Age, years	50–65	5 (55.6)	4 (40.0)	9 (47.4)	4 (33.3)	2 (22.2)	6 (28.6)	15 (37.5)
	66–80	4 (44.4)	6 (60.0)	10 (52.6)	5 (41.7)	7 (77.8)	12 (57.1)	22 (55.0)
	≥ 81	0 (0.0)	0 (0.0)	0 (0.0)	3 (25.0)	0 (0.0)	3 (14.3)	3 (7.5)
Relationship status	Single	1 (11.1)	1 (10.0)	2 (10.5)	2 (16.7)	0 (0.0)	2 (9.5)	4 (10.0)
	Married/co-habiting	1 (11.1)	3 (30.0)	4 (21.1)	2 (16.7)	6 (66.7)	8 (38.1)	12 (30.0)
	Separated/divorced/widowed	6 (66.7)	6 (60.0)	12 (63.2)	8 (66.7)	3 (33.3)	11 (52.4)	23 (57.5)
	Other	1 (11.1)	0 (0.0)	1 (5.3)	0 (0.0)	0 (0.0)	0 (0.0)	1 (2.5)
Highest level of education	University degree or higher	0 (0.0)	0 (0.0)	0 (0.0)	2 (16.7)	0 (0.0)	2 (9.5)	2 (5.0)
	A-levels or equivalent	0 (0.0)	0 (0.0)	0 (0.0)	0 (0.0)	0 (0.0)	0 (0.0)	0 (0.0)
	GCSE	2 (22.2)	0 (0.0)	2 (10.5)	2 (16.7)	2 (22.2)	4 (19.0)	6 (15.0)
	Prefer not to say	0 (0.0)	1 (10.0)	1 (5.3)	2 (16.7)	0 (0.0)	2 (9.5)	3 (7.5)
	Other	7 (77.8)	9 (90.0)	16 (84.2)	6 (50.0)	7 (77.8)	13 (61.9)	29 (72.5)
Number of available vehicles	0	3 (33.3)	1 (10.0)	4 (21.1)	6 (50.0)	4 (44.4)	10 (47.6)	13 (32.5)
	1	3 (33.3)	4 (40.0)	7 (36.8)	4 (33.3)	4 (44.4)	8 (38.1)	16 (40.0)
	2	2 (22.2)	3 (30.0)	5 (26.3)	0 (0.0)	0 (0.0)	0 (0.0)	5 (12.5)
	≥ 3	1 (11.1)	2 (20.0)	3 (15.8)	2 (16.7)	1 (11.1)	3 (14.3)	6 (15.0)
Long-term illness limiting PA^a^	Yes	7 (77.8)	5 (50.0)	12 (63.2)	8 (66.7)	7 (77.8)	15 (71.4)	27 (67.5)

*GCSE* General Certificate of Secondary Education, *PA* Physical activity

^a^Data missing for n = 2 (5.0%) participants

In total, 26 participants (65.0%) were interviewed within 4 focus groups, ranging in size from 5 to 8 participants. All participants present at the third intervention session were invited to participate; no participants declined, and all contributed their views. Focus group discussions lasted approximately 30 min, and participants’ characteristics reflected those of the total sample (Table 4).

**Table 4:** Baseline demographic information for focus group participants and total sample

Variable		Focus group (*n*=26) n (%)	Total sample (*n*=40) n (%)
Age, years	50-65	11 (42.3)	15 (37.5)
	66-80	14 (53.8)	22 (55.0)
	≥81	1 (3.8)	3 (7.5)
Relationship status	Single	3 (11.5)	4 (10.0)
	Married/co-habiting	9 (34.6)	12 (30.0)
	Separated/widowed	13 (50.0)	23 (57.5)
	Other	1 (3.8)	1 (2.5)
Highest level of education	University degree or higher	2 (7.7)	2 (5.0)
	A-levels or equivalent	0 (0.0)	0 (0.0)
	GCSE	4 (15.4)	6 (15.0)
	Prefer not to say	0 (0.0)	3 (7.5)
	Other	20 (76.9)	29 (72.5)
Number of available vehicles	0	8 (30.8)	13 (32.5)
	1	8 (30.8)	16 (40.0)
	2	5 (19.2)	5 (12.5)
	≥3	5 (19.2)	6 (15.0)
Long term illness limiting PA^a^	Yes	20 (76.9)	27 (67.5)

^a^Data missing for *n*=1 (3.8%) participant in focus group and *n*=2 (5.0%) participants in total sample

### Recruitment and retention of participants

All members of the four community groups present at the initial information session were invited to participate; 40 of 46 (87.0%) consented. Reasons for non-participation were health reasons (*n* = 5) and not liking the look of the accelerometer used to measure PA (*n* = 1). Overall, 31 (77.5%) participants attended all three education sessions, with at least two-thirds of enrolled participants present at each education session (session 1, *n* = 36 [90.0%]; session 2, *n* = 31 [77.5%]; session 3, *n* = 32 [80.0%]). However, only three participants (7.5%) accepted the offer of motivational follow-up telephone calls.

### Completion of outcome measures

In total, 11 of 40 (27.5%) participants wore an accelerometer as requested and returned data at all time points: some did not wish to wear the device, and others were absent at the time of its distribution. Valid comparative data, relating to accelerometer wear time ≥ 350 min/day for 3 days, were provided by 20% of participants (*n*=8) (Table 5; Additional file 2).

**Table 5:** Number of participants providing valid accelerometer data and Hospital Anxiety and Depression Scale scores at each measurement time point

Group	Baseline		6 weeks		12 weeks		6 months		All time points	
	Valid PA data n (%)	HADS n (%)	Valid PA data n (%)	HADS n (%)	Valid PA data n (%)	HADS n (%)	Valid PA data n (%)	HADS n (%)	Valid PA data n (%)	HADS n (%)
1 (n = 9)	8/9 (88.9)	9/9 (100.0)	7/9 (77.8)	9/9 (100.0)	6/9 (66.7)	8/9 (88.9)	5/9 (55.6)	9/9 (100.0)	5/9 (55.6)	8/9 (88.9)
2 (n = 10)	9/10 (90.0)	10/10 (100.0)	6/10 (60.0)	8/10 (80.0)	5/10 (50.0)	8/10 (80.0)	1/10 (10.0)	8/10 (80.0)	1/10 (10.0)	6/10 (60.0)
3 (n = 12)	12/12 (100.0)	12/12 (100.0)	8/12 (66.7)	10/12 (83.3)	6/12 (50.0)	7/12 (58.3)	2/12 (16.7)	11/12 (91.7)	2/12 (16.7)	5/12 (41.7)
4 (n = 9)	7/9 (77.8)	9/9 (100.0)	4/9 (44.4)	8/9 (88.9)	2/9 (22.2)	8/9 (88.9)	0/9 (0.0)	7/9 (77.8)	0/9 (0.0)	6/9 (66.7)
Total (n = 40)	36/40 (90.0)	40/40 (100.0)	25/40 (62.5)	35/40 (87.5)	19/40 (47.5)	31/40 (77.5)	8/40 (20.5)	35/40 (87.5)	8/40 (20.0)	25/40 (62.5)

*HADS* Hospital Anxiety and Depression Scale

Negative views of the accelerometer were related to the need to wear the accelerometer on the waist as *“it kept slipping down or slipping up” (G1, 2).* Wrist-worn monitors were suggested by participants as a more acceptable alternative.

However, a small number of participants reported that the act of wearing the monitor was motivating and indirectly provided a reminder to increase their PA:

*“...that wee thing makes you aware you need to move.…” (G4, 25)*

The HADS [41] was completed by 62.5% of participants (*n*=25) at all follow-up time points (Table 5; Additional file 2). No participants declined to complete it: non-completion was due to absence. Focus group participants made no comments regarding the HADS, and none reported having any problems completing it during the sessions.

### Acceptability of intervention

Overall, responses to the exit questionnaire (Table 6) were positive: 85% of participants (*n*=34) were somewhat/very satisfied with their involvement in the study. Further, 85% (*n* = 34) stated they were somewhat/very satisfied with the information they were given about the study (participant information letter and verbal explanation). The majority reported that they would recommend the programme to a friend (77.5%; *n*=31) and would be involved in the programme again (75%, *n*=30).

**Table 6:** Participant responses to exit questionnaire

Question	Response	Immediate intervention			Delayed intervention			
		Group 1 (n = 9)	Group 2 (n = 10)	Total (n = 19)^a^	Group 3 (*n* = 12)	Group 4 (n = 9)	Total^a^ (n = 21)	Total^a^ (n = 40)
		n (%)	n (%)	n (%)	n (%)	n (%)	n (%)	n (%)
Satisfied with involvement in study	Very satisfied	6	7	13	7	6	13	26 (65.0)
	Somewhat satisfied	2	1	3	3	2	5	8 (20.0)
	Neither satisfied nor dissatisfied	1	0	1	1	1	2	3 (7.5)
	Somewhat dissatisfied	0	0	0	0	0	0	0 (0.0)
	Very dissatisfied	0	0	0	0	0	0	0 (0.0)
Satisfied with the advice/information received about this study	Very satisfied	6	7	13	8	8	16	29 (72.5)
	Somewhat satisfied	1	1	2	2	1	3	5 (12.5)
	Neither satisfied nor dissatisfied	0	0	0	0	0	0	0 (0.0)
	Somewhat dissatisfied	0	0	0	0	0	0	0 (0.0)
	Very dissatisfied	0	0	0	0	0	0	0 (0.0)
How helpful was it having a buddy?	Great benefit	6	8	14	7	8	15	29 (72.5)
	Some benefit	2	0	2	2	1	3	5 (12.5)
	No benefit	0	0	0	1	0	1	1 (2.5)
How helpful was the information in the leaflet?	Great benefit	5	6	11	7	6	13	24 (60.0)
	Some benefit	3	2	5	3	3	6	11 (27.5)
	No benefit	0	0	0	1	0	1	1 (2.5)
	Don’t know	0	0	0	0	0	0	0 (0.0)
	Did not read it	0	0	0	0	0	0	0 (0.0)
Would you recommend this programme to a friend?	Yes	7	7	14	9	8	17	31 (77.5)
	No	0	1	1	0	1	1	2 (5.0)
Would you be happy to be involved in this programme again?	Yes	7	7	14	9	7	16	30 (75.0)
	No	1	1	2	0	2	2	4 (10.0)

^a^ Total responses for all questions do not correspond to total number of participants because not all participants provided a response for each question

### Programme components

#### Group sessions and education

The informal delivery of the sessions and group discussion was appreciated. Participants enjoyed *“the laughs” (G1, 2)* and the opportunity to ask questions.

*“...we talked on the day you know, saying, ‘What do you mean by that? Or what can we do?’” (G3, 20)*

Many participants commented that the sessions did not focus on traditional forms of PA, such as running and gym-based activities. They enjoyed hearing about activities that they already engaged in, such as *“hoovering your stairs” (G3, 19)* and that could be easily integrated into their daily life.

*“...you wouldn’t class that as exercise … if you are doing the housework.” (G3, 20)*

#### Printed materials

The information regarding free access to leisure centre facilities for older people at off-peak times was spoken of favourably, but some reported that the times were inconvenient, and there was confusion regarding membership renewal. Although the participants had suggested the walking routes for inclusion in the information pack, some commented that they were not useful as they *“all know the area” (G4, 23)*. It was considered that walks in other geographical areas would be more attractive.

*“...it sometimes can be boring walking in somewhere you know.… It’s interesting walking somewhere where you haven’t seen before.” (G4, 23)*

Most of the maps featured walks in local parks, but participants reported fear of potential anti-social behaviour in those locations. Only one participant felt that parks are relatively safe due to the presence of other park users.

*“The only thing is walking up the park.… We are too frightened to go on our own.” (G2, 11)*

*“...there’s more security ’cause … people can see what’s going on.” (G1, 6)*

#### Buddy support

Whilst most participants in the exit questionnaire stated that having a ‘buddy’ was of some/great benefit, no one in the focus groups had actually identified a ‘buddy’. Reasons included that group members *“don’t live near one another” (G2, 10)*, preferred engaging in PA alone because they *“just like to think” (G1,* 3), and difficulty in identifying an appropriate person.

*“I’ve got my daughter, but I need to get her motivated; she’s always sitting on her phone, you know.” (G1, 3)*

#### Telephone calls

Participants reported that they had declined the offer of follow-up telephone calls because they already received too many telephone calls and had an aversion to answering calls from unknown telephone numbers.

*“Quite often you get that many calls you’re ready for throwing your phone out.” (G3, 20)*

*“If I didn’t recognise the number, I wouldn’t answer.” (G1, 5)*

SMS texts were not considered to be a helpful alternative, owing to difficulties with mobile phones.

*“No, no because I can’t text. There’s no point.” (G2, 10)*

*“I don’t even hear it if you text.” (G2, 10)*

The importance of pre-arranging the timing of telephone calls was highlighted by a participant who engaged in and appreciated follow-up telephone calls:

*“...you asked me what time would suit me. You know really, it was good.” (G2, 12)*

### Suggested improvements for future study

#### Organised group activities

The majority of participants expressed a need for someone to organise activities on their behalf, involving the whole group or conducted during their group session. They felt that this would be enjoyable and allow participants to go at their *“own pace” (G2, 12).* Also of note, it was considered that the opportunity to participate in an organised activity would be more helpful than merely giving information.

*“...if we went as a group. I think that would be good.” (G2, 10)*

*“That would be great craic in here I think. See if we did the exercise here while sitting on a Friday.” (G2, 12)*

*“You could sit and talk about it all you like, but you need to put it into practice.” (G2, 10)*

#### Goal-setting/self-monitoring

Most of the participants who wore the accelerometer expressed a desire for weekly data feedback to support their behaviour change and enable comparison with others in the group.

*“I never heard no feedback, and it frustrated me.” (G2, 10)*

*“...you say next week ‘I’m going to do better, I’m definitely going to do better’. That’s really important.” (G2, 14)*

## Discussion

These findings indicate that delivery of a trial of this PA intervention using pre-existing groups within socio-economically disadvantaged areas is feasible, although refinement of intervention components and methods of delivery is required. The high recruitment and retention rates suggest that tapping into pre-existing social structures may overcome previously reported recruitment and attendance barriers [17, 18, 20, 21, 54]. Data from the focus groups and exit questionnaires suggest that it was an acceptable intervention, and a community centre is an appropriate setting in which to deliver PA education. However, scheduling of sessions and collection of valid objectively measured PA data were challenging.

### Participant recruitment and retention

This study provides evidence that using pre-existing groups for intervention recruitment and delivery may provide a more feasible and sustainable approach than establishing new groups for interventions [33]. Delivering the intervention in venues already used by the target population of women may have helped overcome common barriers to participating in health promotion programmes, such as inconvenience and disruption of their daily life [55]. Recruitment may have been enhanced by social desirability due to visibility as other group members could see if they consented and they were a captive audience. This is of particular importance because socio-economically disadvantaged groups are typically difficult to recruit to PA interventions [23]. However, challenges arose in timing the delivery of intervention content owing to a group changing their schedule, resulting in the need to rearrange sessions and liaise with group leaders. However, flexibility in delivery ensured that groups were satisfied with continued involvement and were not lost to attrition [56].

### Completion of outcome assessments

The poor accelerometer acceptance rate and acquisition of valid objective PA data may be due to a dislike of a waist-worn monitor [57, 58], highlighting the need to consider both accuracy and acceptability issues in selecting PA outcome measures [59]. However, some participants reported that the act of wearing the monitor increased their awareness of needing to increase their PA [60].

The HADS [41] had good completion rates; it was short and easy to complete. The researcher attending the community groups built a rapport with participants, and direct contact with them reduced the potential for loss of questionnaires [56].

### Randomisation and study design

Our initial plan had been to randomly allocate groups to control and intervention conditions, but this was not acceptable to group leaders who considered that the opportunity to receive the intervention should be given to all study participants. Randomisation to a delayed intervention design approach was acceptable, but this led to participants in the delayed intervention groups receiving the intervention during the winter months. They commented that they would have preferred to receive the intervention in better weather. Existing evidence shows that levels of PA vary with seasonality: poor weather, low temperature and short day length are associated with reduced PA [61–63]. Our provision of the information booklet of local opportunities for PA, including activities in community centres and leisure centres, reflects Tucker and Gilliland’s [61] recommendation to provide opportunities for indoor PA during winter months. However, such information was seemingly not used by our participants.

### Theoretical framework

SPT appears to be an appropriate theory for delivering this intervention to promote PA among women in socio-economically disadvantaged communities. The findings support the design of an intervention based on SPT’s three elements of ‘Materials’, ‘Meanings’ and ‘Competencies’. Participants enjoyed the education sessions, which provided practical and emotional support with instruction regarding PA (materials) and social interaction and had potential for health and social consequences (meanings), and many reported improved understanding regarding PA (competencies).

### Acceptability of intervention

Using pre-existing groups may have facilitated group discussions. The women in each group shared similar demographics; information given was appropriate and relevant to their age and ability [55]. Participants could share common experiences, and pre-existing social bonds enabled relaxed conversation [64]. Focus groups revealed the appreciation of information on activities that they could integrate easily into their lives. Older adults commonly engage in PA around and in close proximity to their home [65]. This suggests that future PA promotion interventions should focus on activities of daily living or home-based PA as a sustainable and acceptable source of PA for older adults rather than promoting new recreational activities.

The identification of a friend or relative to engage in PA has been found to be motivating to increase engagement in PA [27, 32, 54, 65] and likely to increase adherence to lifestyle programmes [66, 67]. However, despite encouragement to identify a buddy, none of the focus group participants had done so, although the majority of participants stated in the exit questionnaire that having a buddy was of great benefit. This apparently contradictory finding may be due to misinterpreting the question, questionnaire fatigue or social desirability bias. Reasons for lack of buddy identification may be due to not wanting to be more active, illness, poor understanding of positive lifestyle change, lack of an appropriate person or the influence of negative social norms [30, 68].

Despite the increased popularity of telephone-delivered interventions for health behaviour change, there was a poor uptake of telephone calls, and participants disliked texted prompts due to difficulties using or hearing their mobile phones. This reflects the mixed evidence of the effectiveness of such interventions [69] and highlights that future researchers should be aware of such issues when designing technology-based interventions for older people. Further, the report of already receiving too many telephone calls may reflect the proliferation of telephone ‘cold calling’, potentially limiting the usefulness of telephone calls for intervention follow-up in the future.

In contrast to previous research [29, 70–73], the printed materials in the current study were little used despite participants having been given the opportunity to provide input into their development. Whilst the focus group comments regarding fear of lack of safety when walking in parks concurred with previous reports [31], this was not considered as an issue when requesting information to support their PA, perhaps indicating that they had not seriously considered walking there prior to participation in this study.

### Strengths and limitations

The participants involved in this study were older women living in socio-economically disadvantaged areas, who are typically difficult to recruit [22], may not normally volunteer for research or may not be interested in their health.

Despite accelerometer wear time being low, the collection of valid and objective PA data is preferable to subjective self-report data [76]. The fact that those who wore the accelerometer stated that they would have appreciated feedback of its data is positive information that should inform the choice of devices offered in future work. The HADS [41] had a successful completion rate [77], possibly supported by the researcher having built a rapport with participants and being present to address any difficulties when the questionnaires were being completed. However, her presence may have influenced response bias when completing the exit questionnaires. These included a considerable amount of missing data but were completed at the last session, with no further opportunity for review.

Focus groups were conducted during scheduled group sessions, so that women who had declined to wear an accelerometer were present, allowing insights from participants for whom data collection was incomplete. The sessions also offered a relaxed environment, which may have helped elicit information.

The intervention was developed using the MRC guidelines for the development and evaluation of complex interventions [36] and has reported detail regarding the evidence used, the process of development and the theoretical framework. Transparency in reporting makes interventions more likely to be implemented and may assist others planning similar interventions with scientific rigour [36, 74]. Literature recommends interventions to have a theoretical underpinning [22, 75]; SPT proved to successfully guide the intervention development. However, as well as using SPT as a theoretical framework in intervention development, additional consideration of other approaches with a substantial evidence base for effectiveness in behaviour change may have helped in refining its components.

Although there was input from a community centre staff member and participants into the development of the intervention and the printed materials, it may have been beneficial to increase their involvement and gain it earlier in the process to maximise ownership, refine intervention components and explore strategies to increase usage.

The random allocation with delayed intervention design allowed testing of the intervention in different seasons and enabled all participants to receive the intervention, but increased the labour intensity and cost of printed materials due to the need to deliver the intervention to all groups involved in the study. Difficulties with scheduling also occurred; although the content did not differ, one group received one less group session. This could be considered intervention infidelity, but a single researcher (ERL) delivered the intervention and confirmed that it was delivered as intended and as described in Table 2.

### Recommendations for further study

Accelerometer wear time may be improved by giving data feedback which would support PA goal-setting, an effective and recommended approach for healthy behaviour change [27, 78, 79]. Further consideration of PA measurement tools is required, possibly using wrist-worn rather than waist-worn devices.

Although the group discussion sessions were well received, other social support components should be explored: participants failed to identify PA buddies, but focus groups suggested that organised group activities would be welcomed, potentially overcoming this lack of social support. The likelihood of identifying a PA buddy may also be increased if this is introduced in the first session, rather than in the third session, and repeated in later sessions. Use of a measure for social support may provide further insights into its influence on PA and intervention engagement. Poor uptake of telephone calls also suggests a need for a different follow-up strategy, possibly via email, although further exploration of a preferred method is required. In a larger study in which several individuals may deliver the intervention, fidelity could be facilitated by providing a detailed manual and could be assessed by various methods of observation.

A pilot study of this intervention is required to assess the required effect size and to test if these proposed adaptations to intervention are acceptable and feasible before embarking on a definitive trial. Prior to that pilot, further input from the public and stakeholders should be sought regarding trial design and delivery, particularly with regard to using a parallel-group design in evaluation.

## Conclusions

The high recruitment and retention rates achieved in this study suggest that a trial of this PA intervention is feasible. The intervention content was acceptable to older women living in socio-economically disadvantaged communities, as was recruitment from pre-existing social groups and delivery in community centres. The need for flexible timing of intervention delivery is recognised, with refinement of the intervention design, such as providing accelerometer data feedback to support PA goal-setting and identifying other social support components, including organised group activities. Further consideration of choice of outcome measures, including other objective PA measurement tools and a possible measure of social support, is required. Our findings suggest that a pilot study of a refined intervention should be conducted and may lead to the provision of definitive evidence of the merits of capitalising on existing social structures and using community centres in the promotion of PA for ‘hard to reach’ populations.

## Additional files

Additional file 1: Interview schedule for focus groups. (DOCX 15 kb)

Additional file 2: Consolidated Standards of Reporting Trials (CONSORT) flow diagram of participant recruitment and retention. (DOC 37 kb)

## Abbreviations

BCT: Behaviour change technique
CVD: Cardiovascular disease
HADS: Hospital Anxiety and Depression Scale
MRC: Medical Research Council
PA: Physical activity
SPT: Social Practice Theory
