# Supplementary material for: Promoting physical activity among community groups of older women in socio-economically disadvantaged areas: randomised feasibility study
Source: Trials. 2019 Apr 25;20:234. doi: 10.1186/s13063-019-3312-9 (PMC6482492; doi:10.1186/s13063-019-3312-9)
Supplement: Supplementary file 1 — Interview schedule for focus groups. (DOCX 15 kb) [file 13063_2019_3312_MOESM1_ESM.docx]

Additional file 1; Interview schedule for focus groups

*Location and type of physical activity*

- What types of physical activity did you take part in?
- Where did you do most of your physical activity?

*Barriers and facilitators to physical activity*

- What problems have you experienced whilst trying to be physically active?
- Can you suggest ways you overcame these problems?

*Buddy support*

- Did you use a buddy? (if answer is no then ask: And why?)
- Do you think this buddy support has changed your physical activity levels?

*Map of local opportunities for physical activity*

- Did you use the map of local opportunities for physical activity? (If answer is no then ask: And why?)
- What did you find was the most useful part of the map? And why?
- What did you find was the least useful part of the map? And why?
- How do you think the map could be improved?

*Education sessions*

- What did you find was the most useful part of the education sessions? And why?
- What did you find was the least useful part of the education sessions? And why?
- How do you think the education sessions could be improved?

*Group sessions and telephone calls*

- Do you think having the support of the group changed your physical activity levels? And why?
- Did you receive telephone calls?
- Do you think this telephone support influenced your physical activity levels? And why?

*Overall experience*

- What did you like best about the programme? And why?
- What did you like least about the programme? And why?
- Did you enjoy or dislike taking part in the programme? And why?
- How do you think the programme could be improved?
- How has your level of physical activity changed since the end of the programmes and why?
- Would you be involved in a similar study again?
