# Supplementary material for: Promoting physical activity among community groups of older women in socio-economically disadvantaged areas: randomised feasibility study
Source: Trials. 2019 Apr 25;20:234. doi: 10.1186/s13063-019-3312-9 (PMC6482492; doi:10.1186/s13063-019-3312-9)
Supplement: Supplementary file 2 — Consolidated Standards of Reporting Trials (CONSORT) flow diagram of participant recruitment and retention. (DOC 37 kb) [file 13063_2019_3312_MOESM2_ESM.doc]

Additional file 2; CONSORT flow diagram of participant recruitment and retention

**Baseline**

**Six month follow-up**

**Six week follow-up**

**Enrollment**

**Twelve week follow-up**

Invited to participate in study (n=46)

Declined to participate in study (n=6)

- Health reasons (n=4)
- Not like look of monitor (n=1)
- Wheelchair bound (n=1)
- Completed HADS (n=19/19)
- Number willing to wear accelerometer (n=19/19)

Consented to participate in study (n=40)

- Completed HADS (n=17/19)
  - Absent due to illness (n=2)
- Number willing to wear accelerometer (n=16/19)
  - Absent due to illness (n=2)
  - Health reasons (n=1)
- Completed HADS (n=16/19)
  - Absent due to illness (n=1)
- Number willing to wear accelerometer (n=12/19)
  - Absent due to illness (n=1)
  - Health reasons (n=1)
  - Not want to wear (n=3)
  - Unknown (n=2)
- Completed HADS (n=17/19)
  - Absent due to illness (n=1)
  - Absent due to childcare duties (n=1)
- Number willing to wear accelerometer (n=9/19)
  - Absent due to illness (n=1)
  - Absent due to childcare duties (n=1)
  - Health reasons (n=1)
  - Not want to wear (n=7)

**Immediate Intervention Group (n=19)**

- Completed HADS (n=21/21)
- Number willing to wear accelerometer (n=20/21)
- Completed HADS (n=18/21)
  - Admitted to hospital (n=1)
  - Absent due to holiday (n=2)
- Number willing to wear accelerometer (n=13/21)
  - Admitted to hospital (n=1)
  - Absent due to holiday (n=2)
  - Health reasons (n=1)
  - Not want to wear (n=4)
- Completed HADS (n=15/21)
  - Absent due to illness (n=3)
  - Absent due to holiday (n=2)
  - Admitted to hospital (n=1)
- Number willing to wear accelerometer (n=10/21)
  - Absent due to illness (n=3)
  - Absent due to holiday (n=2)
  - Admitted to hospital (n=1)
  - Health reasons (n=1)
  - Not want to wear (n=4)
- Completed HADS (n=18/21)
  - Absent due to illness (n=1)
  - Absent due to childcare duties (n=1)
  - Unknown (n=1)
- Number willing to wear accelerometer (n=4/21)
  - Absent due to illness (n=1)
  - Absent due to childcare duties (n=1)
  - Health reasons (n=1)
  - Not want to wear (n=13)
  - Unknown (n=1)
  - Not want to wear (n=7)

**Delayed Intervention Group (n=21)**
